# *Gasterocercus depressirostris depressirostris* (Fabricius) (Coleoptera, Curculionidae) – a new xylophagous pest of *Quercus cerris* L. and a new robber fly (Diptera, Asilidae) predator on it in Bulgaria

**DOI:** 10.3897/BDJ.13.e167767

**Published:** 2025-10-06

**Authors:** Veselin Ivanov, Mihail Kechev, Margarita Georgieva, Sevdalin Belilov, Plamen Mirchev, Zdravko Hubenov, Lyubomira Georgieva, Georgi Georgiev

**Affiliations:** 1 Forest Research Institute, Bulgarian Academy of Sciences, Sofia, Bulgaria Forest Research Institute, Bulgarian Academy of Sciences Sofia Bulgaria; 2 National Museum of Natural History, Bulgarian Academy of Sciences, Sofia, Bulgaria National Museum of Natural History, Bulgarian Academy of Sciences Sofia Bulgaria; 3 Sofia University 'St. Kliment Ohridski', Faculty of Geology and Geography, Sofia, Bulgaria Sofia University 'St. Kliment Ohridski', Faculty of Geology and Geography Sofia Bulgaria

**Keywords:** Turkey oak, weevil pest, *Pogonosoma minus*, new records, trophic association, south Dobrudzha

## Abstract

The insect pest complex of Turkey oak (*Quercus cerris* L.) was studied in the field protective forest belts planted on the territory of the State Forest Enterprise Dobrich in south Dobrudzha, north-eastern Bulgaria. Stem cuttings attacked by xylophagous insect pests were collected on 13 March 2025 in the land of Pobeda and Metodievo Villages. The samples were reared in photoeclectors at room temperature (18-22°C). During the period from 24 June to 24 July 2025, the oak weevil *Gasterocercus depressirostris depressirostris* (Fabricius, 1792) (Coleoptera, Curculionidae) was reared for the first time from *Quercus cerris* L. in Bulgaria. Additionally, a new species for Bulgaria's asilid fauna, *Pogonosoma minus* Loew, 1869 (Diptera, Asilidae), was observed developing in the larval galleries of the pest. The trophic relationship between the predatory robber fly and the weevil prey was previously unknown and is recorded for the first time in this study.

## Introduction

In the 1950s, a system of field protective forest belts (FPFBs) was established in Bulgaria to reduce wind erosion, improve soil moisture storage, increase the agricultural crop yields, enhance the environmental microclimate and protect the biodiversity in deforested regions (Georgiev 1960, Marinov et al. 2003, Dodev et al. 2023a). The dominant tree species used for planting were mainly from the genera *Quercus*, *Fraxinus, Gleditsia, Robinia, Juglans* and *Ulmus* (Dodev et al. 2023b).

In 2022, the area of FPFBs in Bulgaria covered 10695.5 ha. The predominant part (72%, or 7668.2 ha) is located in south Dobrudzha, north-eastern Bulgaria, on the territories of the State Hunting Enterprise (SHE) Balchik and the State Forest Enterprises (SFEs) General Toshevo and Dobrich (Mateva and Kirilova 2022).

Since 2020, a dieback process of ash trees (*Fraxinus* spp.) has been observed in the FPFBs in north-eastern Bulgaria (Mateva and Kirilova 2022, Dodev et al. 2023c). During the period 2020–2023, severe damage caused by singing cicadas (Hemiptera, Cicadidae) was registered on ash trees in the FPFBs in south Dobrudza (Georgieva et al. 2024b). Intensive damage by insect pests and fungal pathogens was also identified on other tree species in FPFBs in the region (Georgieva et al. 2024a, Georgieva et al. 2024c).

The present note reports a new xylophagous weevil pest, *Gasterocercus depressirostris depressirostris* (Fabricius, 1792) (Coleoptera, Curculionidae) of *Quercus cerris* L. and a new asilid predator, *Pogonosoma minus* Loew, 1869 (Diptera, Asilidae) on it in the FPFBs in south Dobrudzha in Bulgaria.

## Material and methods

**Figure 1.**
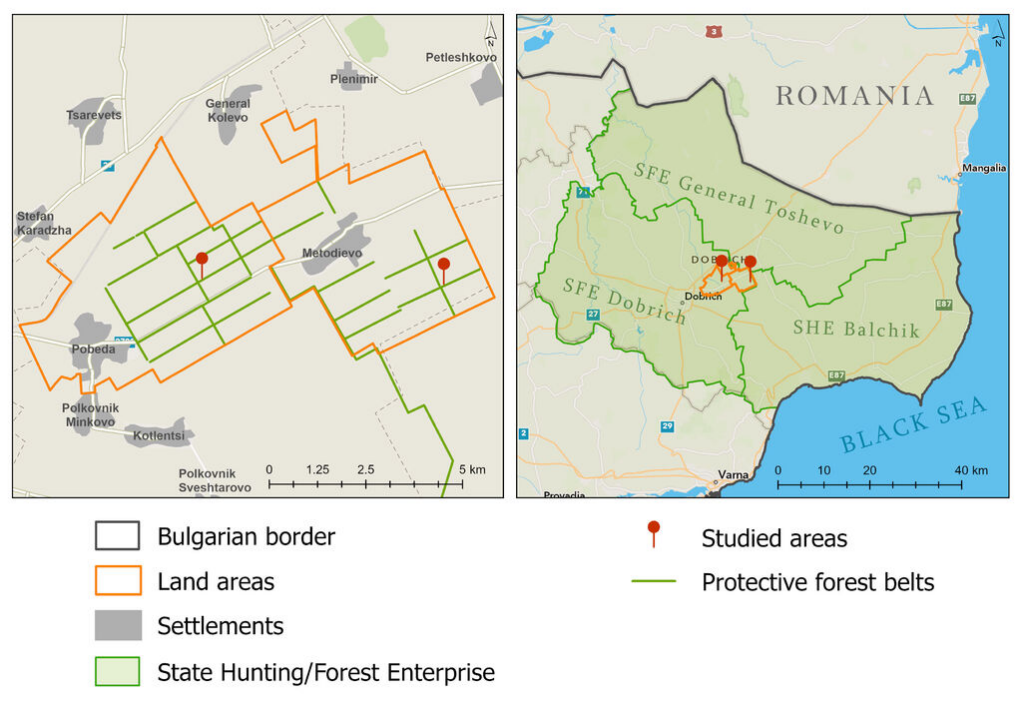
Studied areas in the field protective forest belts on the territory of the State Forest Enterprise Dobrich in south Dobrudzha.

The studies were carried out in 2025 in the outskirts of Pobeda and Metodievo Villages on the territory of SFE Dobrich in south Dobrudzha (Fig. 1). The biological material was collected in FPFBs of *Quercus cerris*. The geographical coordinates, altitudes and other main characteristics of the studied areas are presented in Table 1.

The samples of trees with symptoms of xylophagous pest infestation (stem cuttings with a length of 25 to 30 cm and a diameter of 15 to 25 cm) were taken on 13 March 2025 and transported to the SFE General Toshevo, where each cutting was kept in a separate photoeclector at a temperature of 18-22°C. The samples were observed daily for the emergence of insect pests.

*Gasterocercus depressirostris depressirostris* was identified by the key of Arnoldi et al. (1965). Taxonomic keys of Rihter (1968) and Astakhov (2015)were used for the identification of the species *Pogonosoma minus* and the genus *Pogonosoma* .

The biological material was deposited in the entomological collection of the Forest Research Institute in Sofia.

## Results

In laboratory conditions, two taxa were reared from the stem samples: *Gasterocercus depressirostris depressirostris* (Fabricius, 1792) (Coleoptera, Curculionidae) (Pobeda vill.: 19 ex., 24 June – 04 July 2025; Metodievo vill.: 2 ex., 04 July 2025) and *Pogonosoma minus* Loew, 1869 (Diptera, Asilidae) (Pobeda vill.: 7 ex., 24 June – 24 July 2025; Metodievo vill.: 3 ex., 24 June – 04 July 2025). One of them, *P. minus*, is a new species for the Bulgarian fauna.

**Figure 2.**
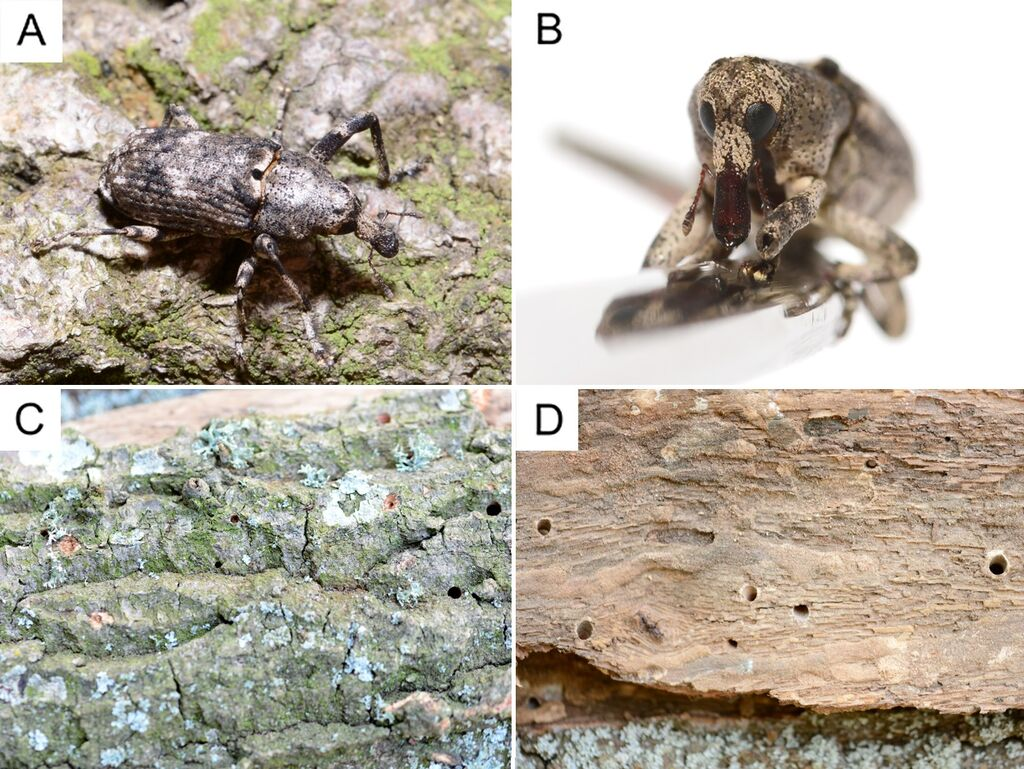
*Gasterocercus depressirostris depressirostris.* **A** adult (dorsal view); **B** head (frontal view); **C** exit holes on bark; **D** larval galleries in the wood.

The adults of *G. depressirostris depressirostris* (Fig. 2A, B) emerged from stems of living *Q. cerris* trees. The exit holes on the bark are round, with a diameter of 6-10 mm (Fig. 2C). The larval galleries penetrate deep into the sapwood (Fig. 2D).

**Figure 3.**
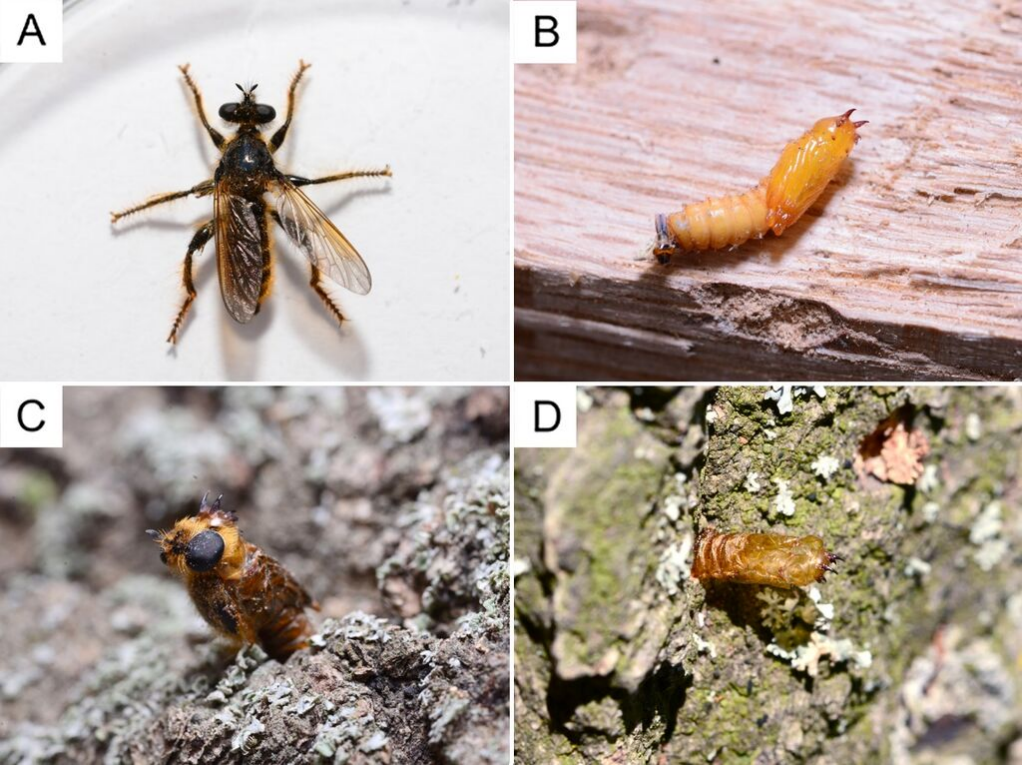
*Pogonosoma minus*: **A** adult (dorsal view); **B** pupa (lateral view); **C** adult emergence from a pupa; **D** empty exuvia.

*Pogonosoma minus* (Fig. 3A) developed within the larval galleries of *G. depressirostris depressirostris*, where the predator’s larvae fed on the weevil. The species’ pupae were located under the bark (Fig. 3B). The emergence occurred through holes in the bark, during which a part of the pupa emerged (Fig. 3C). Part of the exuvia protruded from the bark (Fig. 3D).

The trophic association of *P. minus* with *G. depressirostris depressirostris* was established for the first time in the present study.

## Discussion

The genus *Gasterocercus* Laporte & Brullé, 1828 comprises nine species distributed in the Palaearctic, Oriental and Australian realms, living on dead wood or in dying trees (Alonso-Zarazaga et al. 2009). In Europe, it was described as two species – *G. depressirostris* and *G. hispanicus* Alonso-Zarazaga, Jover & Micó, 2009 (Alonso-Zarazaga et al. 2009). Recently, they were regarded as subspecies of *G. depressirostris* as follows: *G. depressirostris depressirostris*, distributed in Albania, Armenia, Austria, Azerbaijan, Bulgaria, Croatia, Czechia, France, Georgia, Germany, Greece, Hungary, Italy, Moldova, Poland, Romania, Slovakia, Slovenia, South European Russia, Ukraine and *G. depressirostris hispanicus* in Spain (Alonso-Zarazaga 2013, Alonso-Zarazaga et al. 2023). *Gasterocercus depressirostris* was recently reported for the fauna of Switzerland, as well (Germann and Sebastian 2014). In Bulgaria, *G. depressirostris depressirostris* was not known and its presence in the Bulgarian fauna is probably due to unpublished materials in entomological collections.

*Gasterocercus depressirostris* develops under the bark and in living wood of oaks and sometimes causes the death of the attacked trees (Malysheva 1960, Arnoldi et al. 1965). The species, together with representatives of *Agrilus* genus (Coleoptera, Buprestidae), are noted as the leading causes of oak decline (Sallé et al. 2014). According to Bernardinelli and Mossenta (2009), *G. depressirostris* is trophically connected not only with oaks, but also with *Fagus sylvatica* L. and its flight occurs from the end of May to the end of July. Drovenik and Vreš (2012) reported that the species is rare and must be included in the Red List of endangered beetles of Slovenia.

In this study, *P. minus* was found as a new species for the Bulgarian asilid fauna. Until now, only one species of the genus *P. maroccanum* (Fabricius, 1794) is known for the country (Hubenov 2021). *Pogonosoma minus* is a Mediterranean species distributed in southern France, Italy, southern Germany and the Caucasus (Lauterborn 1936). The present finding is the first record of the species on the Balkan Peninsula and south-eastern Europe.

The larvae of the Asilidae family usually live in soil and rotten wood, feeding on eggs, larvae and pupae of other insects (Rihter 1968, Astakhov 2015). *Pogonosoma minus* was reared from old, rotting oak trees in the Upper Rhine Region (Lauterborn 1936). However, the development of the species in larval galleries of boring insects in living wood was recorded for the first time in this investigation.

## Conclusions

The discovery of *G. depressirostris depressirostris* and *P. minus* expands our knowledge of the species composition and ecology of oak xylophagous insects in Bulgaria, enriching the asilid fauna of the country.

## Figures and Tables

**Table 1.** Main characteristics of the studied areas.

№	Locality	Geographical coordinates	Altitude, m	Tree species	Age, years	Average height, m	Average D_1.30_, cm
1	Pobeda Village	43.60779°N, 27.93344°E	245	*Quercus cerris*	70	18	30
2	Metodievo Village	43.60583°N, 28.01094°E	200	*Quercus cerris*	70	17	28

## References

[B13414566] Alonso-Zarazaga AM, Jover T M, Micó E (2009). A new species of the genus *Gasterocercus* (Coleoptera, Curculionidae, Cryptorhynchinae) from the Iberian Peninsula, with notes on the ecology of the genus. Zootaxa.

[B13414575] Alonso-Zarazaga A. M., Löbl I, Smetana A (2013). Catalogue of Palaearctic Coleoptera.

[B13414588] Alonso-Zarazaga MA, Barrios H, Borovec R, Bouchard P, Caldara R, Colonnelli E, Gültekin L, Hlaváč P, Korotyaev P, Lyal CHC, Machado A, Meregalli M, Pierotti H, Ren L, Sánchez-Ruiz M, Sforzi A, Silfverberg H, Skuhrovec J, Trýzna M, Castro AJ Velázquez de, Yunakov NN (2023). Cooperative Catalogue of Palaearctic Coleoptera Curculionea. Second Edition..

[B13414613] Arnoldi LV, Zaslavskii VA, Ter-Minaian ME, Gurieva EL, Krizhanovskii OL (1965). Key to insects of the European part of the USSR.

[B13414626] Astakhov DM, Krivokhatsky VA (2015). Proceedings of the Russian Entomological Society.

[B13414639] Bernardinelli I, Mossenta M (2009). Flight period of *Gasterocercus
depressirostris* in relation to temperature in North-eastern Italy. Bulletin of Insectology.

[B13414648] Dodev Y, Georgiev G, Belilov S, Ivanov V, Georgieva M, Madzhov S, Georgieva L (2023). Creation and management of field protective forest belts in northeastern Bulgaria. Ecologia Balkanica.

[B13414660] Dodev Y, Georgiev G, Georgieva M, Ivanov V, Belilov S, Madzhov S, Georgieva L (2023). Health status of the field protective forest belts in Dobrudzha – results from the monitoring carried out in 2022. Silva Balcanica.

[B13414672] Dodev Y, Georgiev G, Georgieva M, Madzhov S, Belilov S, Ivanov V, Marinkov V, Georgieva L (2023). Main characteristics of the field protective forest belts in northeastern Bulgaria. Forest Science.

[B13414685] Drovenik B, Vreš B (2012). *Gasterocercus
depressirostris* (Fabricius, 1792) (Curculionidea, Coleoptera) new for the fauna of Slovenia. Folia Biologia et Geologica.

[B13414694] Georgieva M, Georgiev G, Ivanov V, Hristova M (2024). First record of *Biscogniauxia
mediterranea* (De Not.) Kuntze on *Quercus
rubra* L. in Bulgaria. Historia Naturalis Bulgarica.

[B13414703] Georgieva M, Gjonov I, Ivanov V, Belilov S, Dodev Y, Georgieva L, Mirchev P, Georgiev G (2024). Damage caused by singing cicadas (Hemiptera: Cicadidae) in the field protective forest belts in South Dobrudzha, Bulgaria. Historia Naturalis Bulgarica.

[B13414716] Georgieva M, Georgiev G, Mirchev P, Ivanov V, Hristova M, Belilov S, Dodev Y, Madzhov S, Georgieva L (2024). Health status deterioration in field-protective forest belts in northeastern Bulgaria. Silva Balcanica.

[B13414730] Georgiev G (1960). Field protective forest belts in the country.

[B13414738] Germann C, Sebastian W (2014). Erstmeldung von *Gasterocercus
depressirostris* (Fabricius, 1792) für die Schweiz (Coleoptera, Curculionidae). Entomo Helvetica.

[B13414747] Hubenov Z (2021). Species composition and distribution of the dipterans (Insecta: Diptera) in Bulgaria.

[B13414755] Lauterborn R (1936). Faunistische Beobachtungen aus dem Gebiete des Oberrheins und des Bodensees. Mitteilungen des Badischen Landesvereins für Naturkunde und Naturschutz in Freiburg im Breisgau.

[B13414764] Malysheva MS (1960). On the biology of the flat-nosed oak weevil - *Gasterocercus
depressirostris*, F. (Coleoptera, Curculionidae). Proceedings of the All-Union Scientific Research Institute of Plant Protection.

[B13414773] Marinov I, Stiptsov V, Genova F (2003). Agroforestry, past, present and future.

[B13414781] Mateva P, Kirilova M (2022). State of the protective forest belts in Dobrudzha. Gora.

[B13414790] Rihter V A (1968). The robber flies (Diptera, Asilidae) of Caucasus.

[B13414798] Sallé A, Parmain G, Nusillard B, Pineau X, Brousse R, Fontaine-Guenel T, Ledet R, Vincent-Barbaroux C, Bouget (2014). Forest decline differentially 1 affects trophic guilds of canopy-dwelling beetles. bioRxiv.

